# Nutritional Status and Nosocomial Infections among Adult Elective Surgery Patients in a Mexican Tertiary Care Hospital

**DOI:** 10.1371/journal.pone.0118980

**Published:** 2015-03-24

**Authors:** Judith Rodríguez-García, Astrid Gamiño-Iriarte, Edel Rafael Rodea-Montero

**Affiliations:** 1 Department of Nutrition, Hospital Regional de Alta Especialidad del Bajío, Blvd. Milenio 130, San Carlos la Roncha, C.P.37660, León, Guanajuato, México; 2 Department of Research, Hospital Regional de Alta Especialidad del Bajío, Blvd. Milenio 130, San Carlos la Roncha, C.P.37660, León, Guanajuato, México; University of Illinois at Chicago, UNITED STATES

## Abstract

**Background:**

Controversy exists as to whether obesity constitutes a risk-factor or a protective-factor for the development of nosocomial Infection (NI). According to the obesity-paradox, there is evidence that moderate obesity is a protective-factor. In Mexico few studies have focused on the nutritional status (NS) distribution in the hospital setting.

**Objectives:**

The aim of this study was to estimate the distribution of NS and the prevalence of nosocomial infection NI among adult elective surgery (ES) patients and to compare the clinical and anthropometric characteristics and length of stays (LOS) between obese and non-obese patients and between patients with and without NI.

**Methods:**

We conducted a cross-sectional study with a sample (n = 82) adult ES patients (21–59 years old) who were recruited from a tertiary-care hospital. The prevalences of each NS category and NI were estimated, the assessments were compared between groups (Mann-Whitney, Chi-squared or the Fisher's-exact-test), and the association between preoperative risk-factors and NI was evaluated using odds ratios.

**Results:**

The distribution of subjects by NS category was: underweight (3.66%), normal-weight (28.05%), overweight (35.36%), and obese (32.93%). The prevalence of NI was 14.63%. The LOS was longer (p<0.001) for the patients who developed NI. The percentages of NI were: 33.3% in underweight, 18.52% in obese, 17.39% in normal-weight, and 6.90% in overweight patients.

**Conclusion:**

The prevalence of overweight and obesity in adult ES patients is high. The highest prevalence of NI occurred in the underweight and obese patients. The presence of NI considerably increased the LOS, resulting in higher medical care costs.

## Introduction

The 2012 National Health and Nutrition Survey data for Mexico estimated a high prevalence (30.6%-49.0%) of overweight (25 kg/m^2^ ≤ BMI < 30 kg/m^2^), a high prevalence (20.4%-47.8%) of obesity (BMI ≥ 30 kg/m^2^), and a low prevalence (1.0% among adults 20–59 years old) of underweight (IMC < 18.5 kg/m^2^) [1]. The global number of surgeries, including elective surgery (ES) and emergency surgery, is estimated to be between 187.2 and 281.2 million cases per year, which represents approximately 1 case for every 25 people [2]. The post-ES complication rate is between 3% and 23% [3,4]. Because of the diversity of ES complications, they have been classified by the type of intervention necessary for treatment [5]. These complications include wound dehiscence, hematoma formation, slow healing, bleeding [6–8] and nosocomial infection (NI) [9]. The identified preoperative risk factors for developing NI include elderly age, long hospital stay prior to surgery, recent weight loss, presence of systemic diseases such as type 2 diabetes mellitus (T2DM) and hypertension (HTN), smoking, American Society of Anesthesiologists physical status (ASA-PS) score, immunosuppression, and altered nutritional status (NS) [10–12]. There is evidence that malnutrition and nutritional alterations are common complications of immune-compromised state and play significant and independent roles in morbidity and mortality [13,14].

Studies that have assessed the NS in ES patients have identified that approximately 2% are underweight, 24% are normal weight, 58% are overweight and 16% are obese [15,16]. Some studies have identified underweight as a risk factor for the development of NI [17–19]. Controversy exists as to whether obesity constitutes a risk factor or a protective factor for the development of NI. Several authors have identified obesity as a risk factor [20–24]. However, according to the obesity paradox, there is evidence that moderate obesity is a protective factor [25]. In other cases, no relationship between NS and the development of NI has been documented [16].

In Mexico, even though the estimated distribution of NS among the general population is known [1], no studies have focused on the NS distribution in the hospital setting. In addition, information exists for NI rates by hospital, but the type of hospital admission is not specified (i.e., ES, emergency surgery or other causes of hospitalization).

The aim of this study was to estimate the distribution of NS and the prevalence of NI among adult ES patients and to compare the clinical and anthropometric characteristics and the length of stays (LOS) between obese and non-obese patients and between patients with NI and patients without NI.

## Patients and Methods

### Patients

We conducted a cross-sectional study with a sample of 82 Mexican-Hispanic adults (males and females; 21–59 years old) who were recruited prospectively between April 23^rd^, 2012 and January 10^th^, 2014 from consecutive ES patients attending the gastrosurgery, neurosurgery and proctology departments at the tertiary care Mexican High-Specialty Regional Bajío Hospital (Hospital Regional de Alta Especialidad del Bajío, HRAEB). located in León City in Guanajuato state (Mexico). Data about systemic diseases (T2DM and HTN) and other relevant conditions (smoking, alcohol consumption) and ASA-PS score [26] were obtained from the clinical record of patients. The exclusion criteria were a recent surgical event, a recent hospitalization, and an immunosuppressive state of pathological (e.g. HIV/AIDS) or pharmacological (e.g. treatment of cancer or following an organ transplantation) etiology. All patients provided signed written consent upon enrollment.

### Ethics statement

The study protocol was reviewed and approved by the Research and Ethics Committees of the HRAEB. Approval numbers: CI-HRAEB-2012–003 and CEI-04–12 respectively.

### Anthropometric assessment

The anthropometric assessment included measures of weight (kg), height (cm), arm circumference (AC, cm), and triceps skin fold thickness (TSF, mm) using the Lohman technique [27]. Recumbent patients were evaluated using the Chumlea formula [28]. All indicators were measured by well-trained HRAEB nutrition staff. Weight was measured with patients wearing light clothes, without shoes and standing upright at the center of the digital scale (Tanita, Tokio, Japan). Height was measured using a stadiometer (Seca, Hamburg, Germany). AC was measured using anthropometric fiberglass tape (Gulick, Gays Mills, USA). TSF was measured using a skin fold caliper (Lange, Ann Arbor, USA). Weight was measured to the nearest 0.1 kg, and height and AC were measured to the nearest 0.1 cm. In addition, the arm muscle area (AMA, cm^2^), which is an index of total skeletal muscle mass, was calculated using the formula $AMA=\frac{{\left(AC-\pi \cdot TSF\right)}^{2}}{4\pi }$, where AC and TSF were measured in centimeters (cm) and adjusted for sex (subtracting 10 or 6.5 cm^2^ for men and women, respectively) [29]. The arm fat area (AFA, cm^2^) was calculated using the formula $AFA=\frac{AC^{2}}{4\pi }-AMA$, where AC and AMA were measured in centimeters (cm) and adjusted for sex (adding 10 or 6.5 cm^2^ for men and women, respectively) [30]. The AMA and AFA percentiles were assessed using standard tables for adults [29].

### Nutritional status

Body mass index (BMI) was calculated as weight (kg) divided by height squared (m^2^). Using the BMI values, the NS categories were determined using the criteria of the World Health Organization (WHO): underweight (BMI < 18.5), normal weight (18.5 ≤ BMI < 25), overweight (25 ≤ BMI <30) and obese (BMI ≥ 30) [31].

### Nosocomial infection

The development of a NI was identified from two sources, patient clinical records and the NI report issued by the HRAEB epidemiology department. The follow-up period was 30 days after ES, which is the recommended duration for identifying NI, according to the Centers for Disease Control and Prevention (CDC) [32]. NI development was classified as present (+) or absent (-), and there were 5 types of NI (surgical wound site, urinary tract, respiratory tract, vascular catheter insertion site, and sepsis), according to the criteria proposed by the WHO and the CDC [33].

### Statistical analysis

All data were analyzed using the statistical software R [34]. Descriptive statistics were calculated for the patients’ clinical characteristics, anthropometric results and LOS. Frequency analysis was used to estimate the prevalences of each NS category and NI. Additionally, the number of patients was totaled for each department and for each sex. These figures were compared between the obese and non-obese patients and between groups with and without NI using the Mann-Whitney U test, the Chi-squared test or the Fisher's exact test, depending on the variable type. The association between preoperative risk factors and NI was evaluated using odds ratios (OR) with their corresponding 95% confidence intervals (CI). Finally, the association between NI and NS was tested using the Chi-squared test. The sample size allowed the detection of a ≥ 5% difference in any assessment (with type I error alpha = 0.05 and type II error beta = 0.80). In all cases, a statistical significance level of alpha = 0.05 was used.

## Results

**Fig 1:**
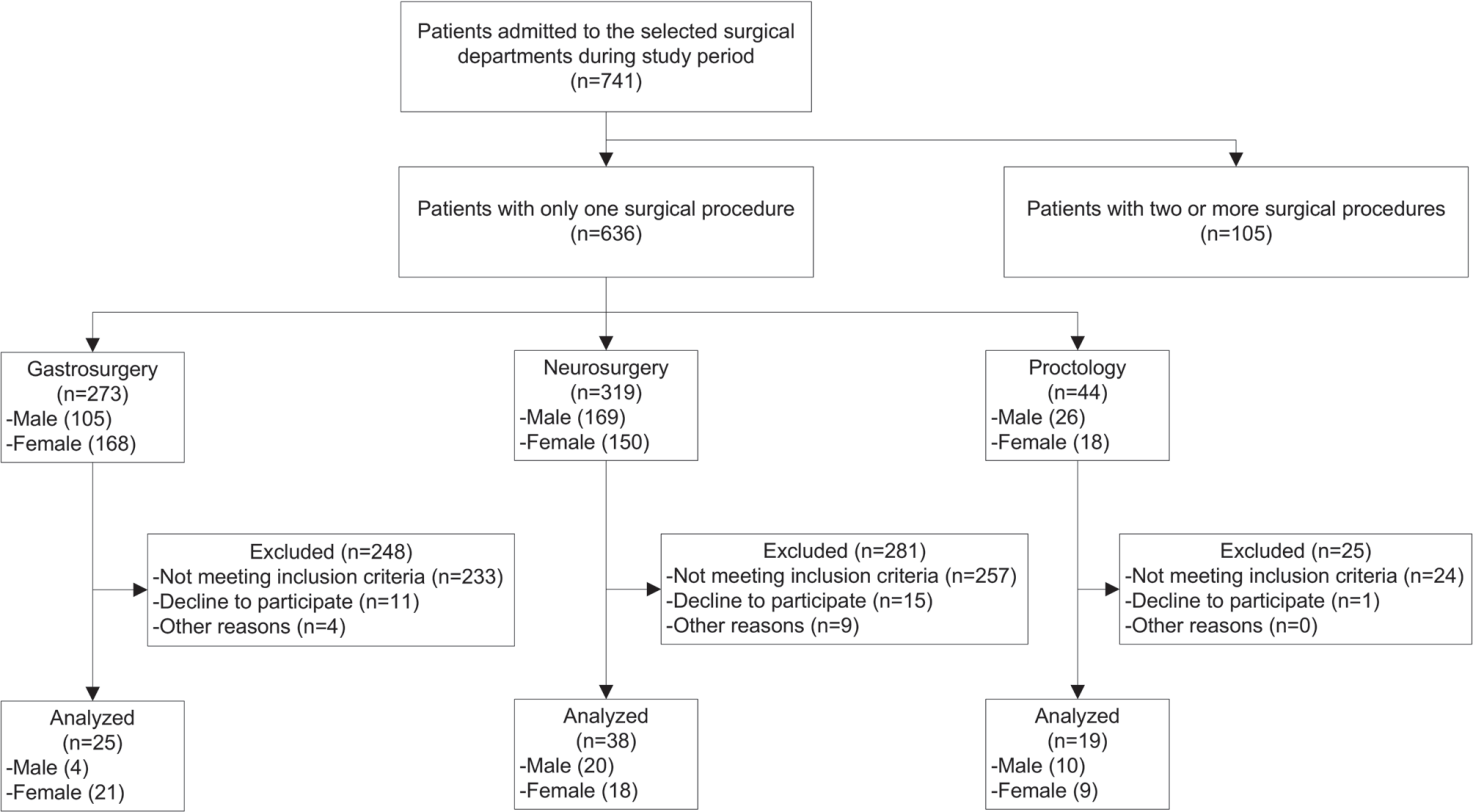
Flow diagram of patient recruitment.

A total of 741 ES patients were admitted to the selected surgical departments during the study period (**Fig. 1**). Of these, 82 ES patients were included in the final analysis, 48 (58.54%) females and 34 (41.46%) males. The mean (± SD) patient age was 41.88 years (± 10.88 years, range 21–59 years). The prevalences of T2DM and HTN in the study population were 7.32% and 19.51% respectively. Among overall patients, 23.17% smoked tobacco and 21.85% drank alcohol. The ASA-PS score distribution in the sample was: ASA-PS I (24.39%), ASA-PS II (65.85%), ASA-PS III (8.54%), ASA-PS IV (1.22%), and there were no patients in the categories ASA-PS V and VI. Using BMI, the distribution of subjects by NS category was as follows: underweight (3.66%), normal weight (28.05%), overweight (35.36%), and obese (32.93%). Overall, the prevalence of NI among the patients was 14.63%, which corresponded to 12 NIs: 4 surgical wound site, 4 sepsis, 2 respiratory tract, 1 urinary tract and 1 vascular catheter insertion site. The mean LOS and the median LOS were 6.54 days and 4 days, respectively (range 2–56 days) (**Table 1**).

**Table 1:** Characteristics of the study population.

	n = 82
Age (years)	41.88 (10.88)
Sex	
Female, n (%)	48 (58.54%)
Male, n (%)	34 (41.46%)
Systemic diseases	
T2DM, n (%)	6 (7.32%)
HTN, n (%)	16 (19.51%)
Relevant conditions	
Smoking, n (%)	19 (23.17%)
Alcohol consumption, n (%)	18 (21.85%)
Anthropometric	
Weight (kg)	71.57 (14.88)
Height (cm)	160.12 (9.99)
BMI (kg/m^2^)	27.85 (5.15)
AMA (cm^2^)	44.45 (13.5)
AMA percentile	56.26 (30.63)
AFA (cm^2^)	28.45 (13.43)
AFA percentile	54.94 (27.3)
Nutritional Status	
Underweight, n (%)	3 (3.66%)
Normal weight, n (%)	23 (28.05%)
Overweight, n (%)	29 (35.36%)
Obese, n (%)	27(32.93%)
Hospitalization	
ASA-PS score (I-VI)	
I, n (%)	20 (24.39%)
II, n (%)	54 (65.85%)
III, n (%)	7 (8.54%)
IV, n (%)	1 (1.22%)
Length of stay (days)	6.54 (8.53)
Nosocomial Infection	
Without Infection, n (%)	70 (85.37%)
With Infection, n (%)	12 (14.63%)

Unless otherwise indicated, the values are given as the mean (S.D.)

The distribution of ES patients by surgical department was gastrosurgery (30.49%), neurosurgery (46.34%), and proctology (23.17%). The percentages of female patients by surgical department were 84% (21/25) in gastrosurgery, 47.36% (18/38) in neurosurgery, and 47.36% (9/19) in proctology. The total number of obese and non-obese patients by department and the total number of patients with and without NI by department are detailed in **S1 Fig**. There was no significant association between surgical department and obesity (p = 0.453). However, a significant association was detected between the surgical department and presence of NI (p = 0.013).

Next, the patients were divided into 2 groups based on BMI, obese (BMI ≥ 30) and non-obese (BMI < 30). Comparing the two groups, age was significantly older and weight was significantly greater in the obese patient group (p < 0.001 in both cases). Regarding body stores, the AMA and AFA values were significantly greater (p < 0.001 in both cases) in the group of obese patients. The presence of HTN was significantly higher (p < 0.027) for the obese patients. There were no significant differences among the groups for any of the following variables: T2DM (p = 0.390), smoking (p = 0.887), alcohol consumption (p = 0.967), and ASA-PS score (p = 0.107). The presence of NI and LOS were similar in the obese and non-obese patient groups (p = 0.518 and p = 0.924, respectively).

**Table 2** shows the characteristics of the study population grouped by the presence or absence of NI. Comparing the two groups, statistically significant differences were detected for sex (p = 0.012), height (p = 0.037), and AMA (p = 0.019). The LOS was significantly longer (p < 0.001) for the patients who developed NI, 4.41 days in patients without NI and 18.92 days in patients with NI. Note that 11 of the 12 NIs occurred in females.

**Table 2:** Characteristics of the study population grouped by the presence of NI.

	Nosocomial Infection (+) (n = 12)	Nosocomial Infection (-) (n = 70)	Significance
Age (years)	42.92 (11.38)	41.7 (10.87)	p = 0.713^a^
Sex			**p = 0.012** ^b^ ^,^ ^c^
Female, n (%)	11 (91.67%)	37 (52.86%)	
Male, n (%)	1 (8.33%)	33 (47.14%)	
Systemic diseases			
T2DM, n (%)	2 (16.66%)	4 (5.71%)	p = 0.211^b^
HTN, n (%)	2 (16.66%)	14 (20.00%)	p = 0.999^b^
Relevant conditions			
Smoking, n (%)	1 (8.33%)	18 (25.71%)	p = 0.278^b^
Alcohol consumption, n (%)	1 (8.33%)	17 (24.29%)	p = 0.286^b^
Anthropometric			
Weight (kg)	64.7 (18.86)	72.75 (13.91)	p = 0.122^a^
Height (cm)	154.42 (10.68)	161.10 (9.61)	**p = 0.037** ^a^ ^,^ ^c^
BMI (kg/m^2^)	26.87 (6.32)	28.02 (4.95)	p = 0.573^a^
AMA (cm^2^)	35.79 (13.29)	45.93 (13.05)	**p = 0.019** ^a^ ^,^ ^c^
AMA percentile	55.75 (33.82)	56.34 (30.31)	p = 0.937^a^
AFA (cm^2^)	31.14 (14.25)	27.98 (13.34)	p = 0.409^a^
AFA percentile	49.33 (30.73)	55.9 (26.79)	p = 0.511^a^
Nutritional Status			p = 0.518^b^
Non-obese, n (%)	7 (58.33%)	48 (68.57%)	
Obese, n (%)	5 (41.67%)	22 (31.43%)	
Hospitalization			
ASA-PS score (I-VI)			p = 0.332^b^
I and II (low risk), n (%)	10 (83.33%)	64 (91.43%)	
III and IV (high risk), n (%)	2 (16.66%)	6 (8.57%)	
Length of stay (days)	18.92 (16.33)	4.41 (3.41)	**p < 0.001** ^a^ ^,^ ^c^

Unless otherwise indicated, the values are given as the mean (S.D.).

^a^Mann-Whitney U test.

^b^Fisher's exact test.

^c^Significant p-values.

The odds of showing NI according to one preoperative clinical feature considered are detailed in **Table 3**. In the bivariate analysis, there was no statistical significance for the OR of any of the following preoperative clinical features: T2DM, HTN, smoking, alcohol consumption, obesity and ASA-PS score.

**Table 3:** Odds ratio to have NI in subjects with one preoperative clinical feature considered.

	OR	95% CI	Significance
Nosocomial Infection			
T2DM	3.3	0.53–20.43	p = 0.199
HTN	0.8	0.16–4.07	p = 0.788
Smoking	0.26	0.03–2.18	p = 0.216
Alcohol consumption	0.28	0.03–2.36	p = 0.244
Obesity	1.56	0.44–5.46	p = 0.488
ASA-PS score (III and IV)	2.13	0.38–12.08	p = 0.392

OR: Odds ratio, CI: Confidence interval.

**Fig 2:**
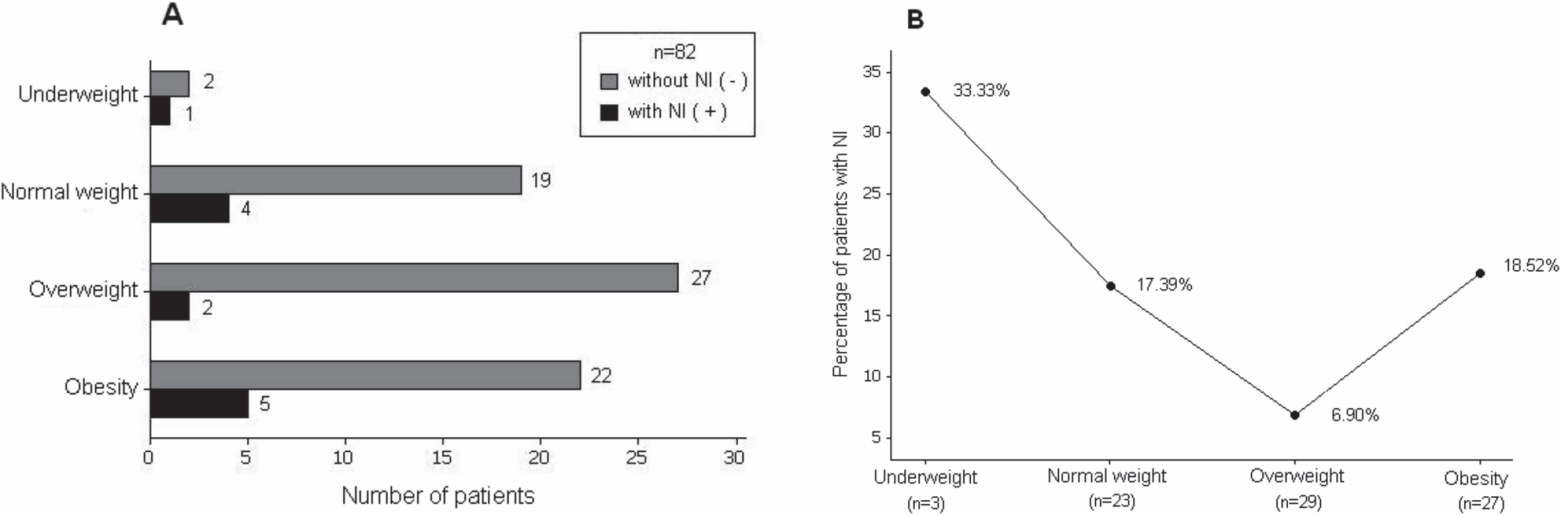
Patients grouped by NS, according to BMI: Panel A: The association between NS and the presence or absence of NI (χ^2^ = 2.696, g.l. = 3, p = 0.441). Panel B: The percentage of patients with NI by NS, based on BMI.

**Fig. 2** shows the total number of patients with and without NI and the corresponding percentages grouped by NS. There was a noticeable but not statistically significant association between NS and the presence of NI (p = 0.441). In descending order, the following percentages of NI were observed: 33.3% in underweight patients, 18.52% in obese patients, 17.39% in normal weight patients, and 6.90% in overweight patients.

## Discussion

Our study only evaluated adult ES patients. The estimated distribution of NS by category was 3.66% underweight, 28.05% normal weight, 35.37% overweight, and 32.93% obese. The obtained low prevalence of underweight could be related to the low prevalence (1.0% among adults 20–59 years old) of underweight estimated by the 2012 National Health and Nutrition Survey data for Mexico [1]. We observed results similar to those described by Mullen *et al*. [25], who identified that patients undergoing non-bariatric general surgery included 2.6% underweight, 30.5% normal weight, 32% overweight and 34.9% obese.

The estimated prevalence of NI was 14.63%; note that patients were recruited from the gastrosurgery, neurosurgery and proctology departments in approximately the same proportion. In our study, the estimated prevalence of NI was lower than the 24.3% prevalence described by Canturk *et al*. [15] but similar to the 13.3% prevalence reported by Pessaux *et al*. [19]. The presence of NI was significantly associated with sex, with 11 of the 12 NIs occurring in females, a result that was similar to that described by Paillaud *et al*. in elderly ES patients [18]. In contrast, Font-Viszcarra *et al*. [17] found no difference in the development of NI by sex, but their study included only patients with total knee replacements. We detected a statistically significant association between the surgical department and presence of NI (p = 0.013), but the low prevalence of NI must be considered when evaluating this result. Patients in the gastrosurgery department had a higher prevalence of NI, which may be because abdominal surgery is identified as a risk factor for the development of NI [35,36] or could be related to the higher female percentage in gastrosurgery (84%) compared with female percentages in neurosurgery and proctology (47.37% in both cases).

In our study, the LOS was not statistical significant different between obese and non obese patients, which agrees with data reported by Alemeida *et al*. [37]. The LOS was significantly longer in patients with NI, the patients who developed a NI had a LOS that was 14.51 days longer than the patients who did not develop NI (p < 0.001). This result is consistent with that reported by Angeles-Garay *et al*. [38] and considering the concept of prolonged stay [39,40] our results suggest that the presence of NI generates a prolonged stay in adult ES patients, similar to the results by Erbaydar *et al* [41].

With regard to the relationship between NS and NI, despite statistical significance for association was not obtained, most likely because of the low prevalence of NI. In our study, a reverse ‘J-shaped’ outcome curve according to NS for adult ES patients may actually exist, in which NI percentage is greatest in underweight patients, which is consistent with Pessaux *et al*. [19]; lowest in overweight, and mildly in normal weight and obese patients similar to Choban *et al*. [20] and Dindo *et al*. [16]. This finding supports the obesity paradox [25] in adult ES patients considering percentage of NI. We did not consider a group of severely obese patients (BMI ≥ 40) because this condition was presented only in one patient of the sample. However, our estimated reverse ‘J-shaped’ curve is similar to the ‘U-shaped curve’ with lowest mortality rates occurring at intermediate BMIs, described by Childers *et al*. [42].

This study has certain limitations. First, this study was cross-sectional in nature, and therefore, causality cannot be inferred. Second, the study sample consisted only of Mexican-Hispanic adults, which may limit generalizability to other ethnic groups. Third, the findings are based on a very small number (n = 12) of patients with NI, thus interpretation should be careful. Last, confounding factors, such as surgical technique, wound care, use of prophylactic antibiotics, and intensive care or mechanical ventilation needs, must be considered as possible contributors to the development of NI. The strengths of study include its prospective design, the adult ES patient (21–59 years old) population, the restriction to 3 surgical departments, and the performance of anthropometric assessments by trained, standardized personnel.

In Mexico, the prevalences of obesity in the general hospital setting and in the specific ES patient group are unknown. Multicentric studies are needed to help estimate the prevalence of obesity in ES patients in Mexico. In addition, studies that use measures of abdominal and visceral fat are also necessary because studies have found these measures to be associated with altered immune function [43,44].

## Conclusions

Our study has expanded the knowledge of the epidemiology of NS and NI and demonstrated a high prevalence of overweight and obesity in adult ES patients in the Mexican-Hispanic population at a tertiary-care hospital. We identified that the highest prevalence of NI occurred in the underweight and obese patients. We also noticed that the presence of NI considerably increased the LOS, thus resulting in higher medical care costs. Further prospective studies assessing the impact of NS in patients with NI that include diverse types of surgical patients, as well as pediatric and elderly populations are needed to more conclusively determine whether the obesity paradox truly exists on ES patients in order to improve clinical decision-making and resource management to prevent adverse events in ES patients.

## Supporting Information

S1 Fig Patients by surgical department: Panel A: The association between the surgical department and the presence or absence of obesity (*χ* ^2^ = 1.584, g.l. = 2, p = 0.453), Panel B: The association between the surgical department and the presence or absence of NI (*χ* ^2^ = 8.752, g.l. = 2, p = 0.013). (TIF)
