# Supplementary figures and images for: Nutritional Status and Nosocomial Infections among Adult Elective Surgery Patients in a Mexican Tertiary Care Hospital
Source: PLoS One. 2015 Mar 24;10(3):e0118980. doi: 10.1371/journal.pone.0118980 (PMC4372354; doi:10.1371/journal.pone.0118980)

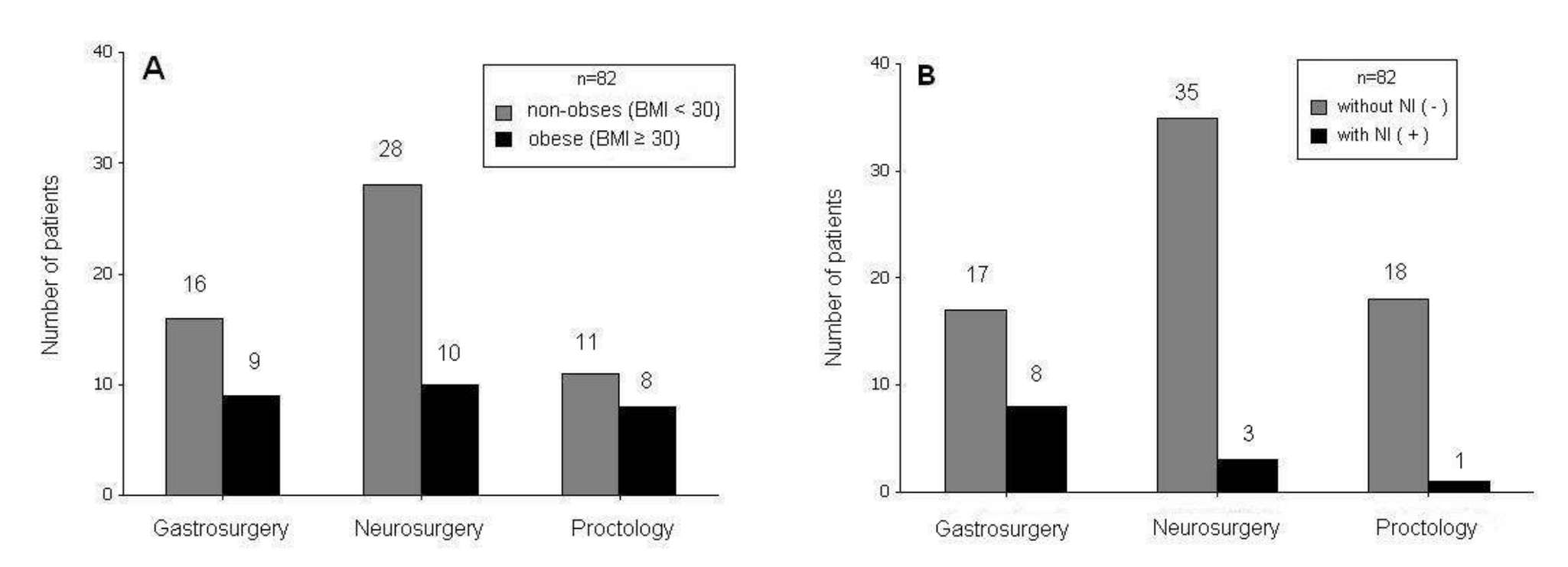

Supplement: S1 Fig — (TIF) [file pone.0118980.s001.tif]
